# The Role of Nontuberculous Mycobacteria in Patients With Cystic Fibrosis Advanced Lung Disease

**DOI:** 10.1111/tid.70190

**Published:** 2026-02-25

**Authors:** Shirin Barcikowski, Ribal Bou Mjahed, Oriol Manuel, Macé M. Schuurmans, Adrian Alonso, Zisis Balmpouzis, Jesica Mazza‐Stalder, Angela Koutsokera, Nicolas J. Mueller

**Affiliations:** ^1^ Department of Infectious Diseases and Hospital Epidemiology University Hospital Zurich University of Zurich Zurich Switzerland; ^2^ Infectious Diseases Service and Transplantation Center Lausanne University Hospital University of Lausanne Lausanne Switzerland; ^3^ Department of Pulmonology University Hospital Zurich University of Zurich Zurich Switzerland; ^4^ Lung Transplantation Center Division of Pulmonology Department of Medicine Lausanne University Hospital University of Lausanne Lausanne Switzerland; ^5^ Adult Cystic Fibrosis and CFTR‐related Disorders Center Division of Pulmonology Department of Medicine Lausanne University Hospital University of Lausanne Lausanne Switzerland

**Keywords:** cystic fibrosis, lung transplantation, nontuberculous mycobacteria

## Abstract

**Background:**

Despite decreased morbidity and mortality in patients with cystic fibrosis (CF) with CFTR modulators, a significant percentage still develop advanced lung disease. Nontuberculous mycobacteria (NTM) remain challenging pathogens with unclear effects on disease progression and post‐transplant outcome.

**Methods:**

NTM‐positive patients were identified from all CF patients treated at the University Hospitals of Zurich and Lausanne between 1998 and 2020. Disease course pre‐ and post‐transplant was recorded, and NTM‐positive patients were compared to patients without NTM infection for death or lung transplantation, as well as post‐transplant survival and development of chronic lung allograft dysfunction (CLAD).

**Results:**

Of 270 patients, 56 (20.7%) met criteria for NTM positivity (two independent cultures with the same organism), with Mycobacterium *abscessus* complex (MABSC) being the most common species (75.0%). Death or transplantation occurred in 12 of 56 (21.4%) NTM‐positive patients and 92 of 198 (46.5%) NTM‐negative patients (OR 3.17; 95% CI, 1.53–7.00; *p* = 0.001). Post‐transplant mortality was higher in the NTM‐positive cohort (3 (27.3%) vs. 12 (13.0%); HR, 4.01, 95% CI, 1.08–14.86; *p* = 0.024), while CLAD incidence did not differ significantly.

**Conclusion:**

NTM‐positive CF patients were associated with a lower pre‐transplant risk of death or transplantation. Post‐transplant mortality was higher in NTM‐positive patients, while no association between NTM infection and CLAD was observed. These findings indicate that favorable outcomes after lung transplantation are achievable in NTM‐positive patients. Thus, NTM‐infection should not be an absolute contraindication for lung transplantation, though careful individual assessment is essential due to the risk of serious complications.

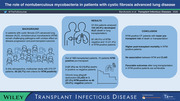

## Graphical abstract

Among 270 cystic fibrosis patients, 20.7% were NTM‐positive and had a lower pre‐transplant risk of death or lung transplantation but higher post‐transplant mortality, with no difference in CLAD incidence. These results suggest NTM infection should not be an absolute contraindication to lung transplantation, though careful individual risk assessment is required.

## 1 Introduction

The prevalence of nontuberculous mycobacteria (NTM) as a cause of clinically relevant infections has increased overall. This increase has been particularly observed in individuals with cystic fibrosis (CF), especially in the pre‐CFTR modulator era [1, 2, 3, 4]. NTM are divided into two groups: slowly growing mycobacteria, including *Mycobacterium avium* complex (MAC), *Mycobacterium kansasii*, and *Mycobacterium simiae* as the most clinically relevant species, and rapidly growing mycobacteria, such as *Mycobacterium abscessus complex* (MABSC, with three subspecies [5, 6]), as well as *Mycobacterium cholenae* and *Mycobacterium fortuitum*. Among the rapidly growing mycobacteria, MABSC species are particularly pathogenic for CF patients due to their natural resilience and ability to develop multiple drug resistance [7]. Current treatment for NTM involves prolonged multi‐drug regimens, which can be associated with severe toxicities [8, 9]. Despite treatment, chronic lung infection with MABSC rarely results in sustained sputum conversion in CF patients [1]. However, the clinical significance of NTM‐positive cultures in CF patients is still unclear [3, 10], as are possible risk factors for acquisition [4].

Although morbidity and mortality have significantly decreased in the era of CFTR modulators, the CF Foundation registry reports that 12% of CF patients have positive cultures for NTM. In addition, about 10% of CF patients have advanced lung disease, defined here as FEV1 less than 40% predicted, use of supplemental oxygen, or other clinical signs of advanced lung disease [11]. In this population, NTM infections may increase morbidity and mortality, both before and after lung transplantation. However, existing literature offers limited insights into the role of NTM infection on disease progression and post‐transplant outcomes. Although still clinically relevant, this subject may become more challenging to study in the CFTR‐modulator era. Lung transplantation waiting list management varies among different transplantation centers, with some centers considering ongoing NTM infection a contraindication for lung transplantation [12, 13], whereas others do not. In the lung transplantation centers of Switzerland (including the University Hospital of Zurich [USZ] and University Hospital of Lausanne [CHUV]), NTM infection is not considered an absolute contraindication for lung transplantation.

The objective of this study is to describe the extent of NTM infection in all CF patients at the USZ and the CHUV CF centers. We also evaluated risk factors for NTM acquisition and the impact of chronic infection on pulmonary disease progression in patients from the pre‐CFTR modulator era. Moreover, we aimed to clarify the significance of NTM infection on post‐transplant outcomes and to describe potential risk factors for ongoing active infection after lung transplantation.

## 2 Methods

### 2.1 Study Design and Patient Characteristics

This retrospective, comparative, multi‐center study was conducted at the USZ and the CHUV, Switzerland. Eligible for inclusion were all CF patients treated at either the Zurich or Lausanne Adult CF centers who were at least 16 years of age. The clinical data were gathered from January 1998 to December 2020 from hospital electronic medical charts. Patients underwent regular clinical assessments every three months or annually, including screening for NTM and other pathogens, using sputum, bronchoalveolar lavage (BAL), or throat swab samples. For transplanted CF patients, NTM screening was carried out during surveillance bronchoscopies. Microbiological data were processed and recorded at the Institute of Medical Microbiology (IMM), National Reference Center for Mycobacteria, University of Zurich, Switzerland. Susceptibility testing was performed for clinically relevant MABSC‐positive sputum samples. For susceptibility analysis, only data from the ZH center were used.

The Cantonal Ethics Committee Zurich and Vaud approved the protocol (Reference: BASEC‐Nr. 2018‐00400). All patients provided a written informed general consent.

### 2.2 Definitions

Patients were defined as NTM‐positive when they had at least two independent positive cultures of an identical NTM species, from lower respiratory tract samples—defined as BAL or bronchial secretions, during the observation period. In the case of transplanted patients, NTM positivity at the time of transplant was used as a reference time point. NTM‐associated pulmonary and extrapulmonary complications following lung transplantation were collected. Chronic infection with *Pseudomonas aeruginosa* was defined as regular positive culture from sputum or respiratory secretions, on two or more occasions extending over six months [14]. Advanced lung disease was defined as death or lung transplantation, whichever came first.

For MABSC NTM‐positive patients, antibiotic susceptibility was recorded. Antibiotic susceptibility testing was interpreted according to CLSI guidelines M24‐A2 [15]. Treatment consisted of combination therapy with at least three susceptible substances. While specific treatment was not recorded, NTM treatment was based on current guidelines, and after consulting an NTM specialist at each center. Indication for treatment was deterioration of lung function or in anticipation of lung transplantation.

### 2.3 Microbiological Methods

All specimens underwent thorough examination for the presence of mycobacteria through fluorescence microscopy and confirmation by Ziehl–Neelsen stain. Cultures were predominantly conducted in a modified Middlebrook 7H9 liquid medium (commercial MGIT, BD, Allschwil, Switzerland) in combination with a solid medium (Middlebrook 7H10/7H11). Positive cultures were then identified for species by DNA‐sequencing (16S rRNA gene, *rpoB* gene, *hsp* 65 kD). Susceptibility testing for MABSC followed CLSI guidelines in a microdilution format [15].

### 2.4 Outcomes

The primary outcome was the progression to death or transplantation in CF patients, defined above as death or the need for lung transplantation. Within the subgroup of patients undergoing lung transplantation, the primary outcome included death or re‐transplantation. Secondary outcomes were NTM‐associated pulmonary or extrapulmonary complications and the development of chronic lung allograft dysfunction (CLAD).

### 2.5 Statistical Analysis

Categorical variables were summarized as frequencies and percentages, and continuous variables as medians and interquartile ranges. *T*‐test and ANOVA variance analysis were used for between‐group comparison of parametric continuous variables; Mann–Whitney and Kruskal–Wallis for non‐parametric variables. Categorical variables were compared using chi‐square tests or Fisher's exact test for small sample sizes. A *p*‐value of 0.05 was considered to indicate statistical significance. All data were analyzed with R software, version 4.2.2 (R Foundation for Statistical Computing).

For the primary outcome (lung transplantation and mortality as a composite and as separate outcomes), we calculated odds ratios and 95% confidence intervals. Mortality following lung transplantation was assessed using log‐rank survival analysis. Cox regression modeling explored the association between survival time and predictor variables. Hazard ratios were interpreted as the likelihood of an individual in the NTM‐positive group experiencing mortality after lung transplantation compared with the NTM‐negative group, with 95% confidence intervals estimated for these outcomes.

## 3 Results

### 3.1 Characteristics of the Patients

**FIGURE 1:**
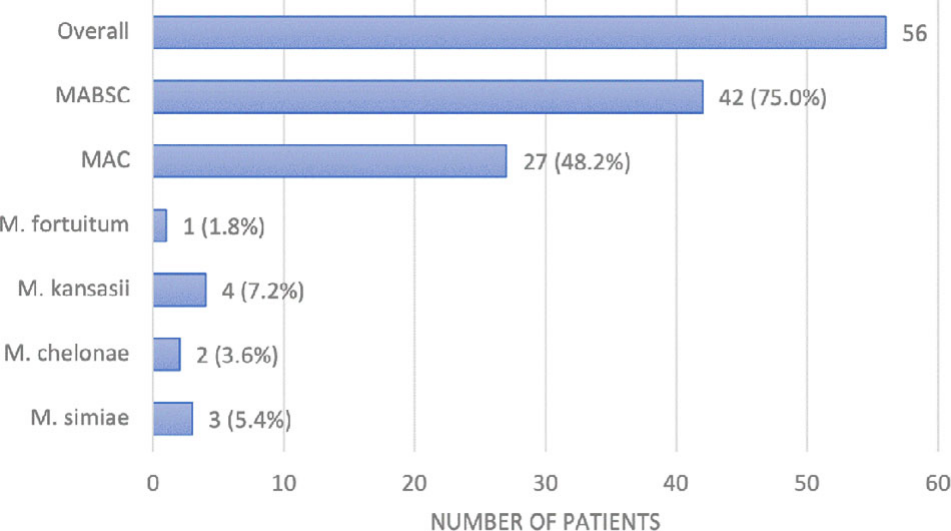
Distribution of NTM species isolated in the total of 56 NTM‐positive cultures. MABSC was isolated in 42 (75.0%) and MAC in 27 (48.2%) cultures. MABSC, *M. abscessus complex*; MAC, *M. avium* complex; NTM, nontuberculous mycobacteria.

From a total of 273 CF patients treated at the Zurich and Lausanne CF centers during the period 1998–2021, three patients who had not provided the general consent were excluded. In addition, sixteen patients who grew only a single NTM‐positive culture of the same NTM species were also excluded. Among the 254 patients analyzed, 56 (22.0%) met the criteria for NTM positivity, defined as having two independent cultures with the same organism. MABSC was the most common species identified in 42 (75.0%) patients, followed by MAC in 27 (48.2%) patients. Notably, 16 (28.6%) patients grew both MABSC and MAC. Species distribution is shown in Figure 1.

The median patient age was 29 years (interquartile range, 26.0–41.0), and 58.9% were male. Concomitant chronic *P. aeruginosa* infection was detected in 47 patients (83.9%). Regarding maintenance therapy, 21 patients (37.5%) of the NTM‐positive group, compared to 105 (53.0%) of NTM‐negative patients, were on long‐term azithromycin treatment three times per week for its immunomodulatory effect (*p* = 0.047). Baseline characteristics of the patients are summarized in Table 1.

**TABLE 1:** Demographics of the CF population and comparison of NTM‐positive to NTM‐negative cases within the CF population (defined as at least two independent confirmed positive cultures of an identical NTM species).

	Overall *n = *254	NTM negative *n = *198	NTM positive (≥ 2 pos) *n = *56	*p*‐value	OR	95% CI
Male (%)	136 (53.5)	103 (52.0)	33 (58.9)	0.445		
Median age (years, [IQR])	33.00 [26.00, 41.00]	33.00 [26.00, 41.00]	29.00 [26.00, 41.00]	0.747		
Mutation^a^ (%)				0.65		
Other mutation	31 (12.2)	26 (13.1)	5 (8.9)			
Heterozygous for F508del	95 (37.4)	70 (35.4)	25 (44.6)			
Homozygous for F508del	108 (42.5)	82 (41.4)	26 (46.4)			
Transplanted (%)	103 (40.6)	91 (46.0)	12 (21.4)	0.001	3.11	1.50–6.86
Pancreatic insufficiency (%)	223 (87.8)	173 (87.4)	50 (89.3)	0.877		
Oral steroid treatment^b^ (%)	83 (32.7)	69 (34.8)	14 (25.0)	0.22		
Chronic *Pseudomonas aeruginosa* infection (%)	196 (77.2)	149 (75.3)	47 (83.9)	0.236		
*Burkholderia cepacia* complex infection (%)	25 (9.8)	19 (9.6)	6 (10.7)	0.801		
Long‐term azithromycin treatment (%)	126 (50.0)	105 (53.0)	21 (37.5)	0.047		
Advanced lung disease (%)	104 (40.9)	92 (46.5)	12 (21.4)	0.001	3.17	1.53–7.00
Death pre‐lung transplantation (%)	1 (0.39)	1 (0.5)	0 (0.0)	1		
Death (pre and post lung transplantation) (%)	16 (6.3)	13 (6.6)	3 (5.4)	1	1.24	0.32–7.04

Abbreviations: CF, cystic fibrosis; NTM, nontuberculous mycobacteria.

^a^ Information on mutation was available in 234, 92.1% of patients.

^b^ Defined as at least one course of oral corticosteroid therapy administered at any time during the observation period.

The subgroup of MABSC‐positive patients was younger at the time of inclusion in study compared with both NTM‐negative (28 years, interquartile range, 24.50–36.50, vs. 33 years, interquartile range, 26–41; *p* = 0.057) or patients with non‐MABSC (41 years, interquartile range, 32–54; *p* = 0.008) (Tables S1 and S2).

They also had acquired NTM at an earlier age (19.5 years, interquartile range, 16.75–32.75, vs. 34 years, interquartile range, 25–39; *p* = 0.015) than patients with non‐MABSC (Table S2). There was no significant difference in the remaining demographic variables.

### 3.2 Advanced Lung Disease in the CF Population

Out of 254 CF patients analyzed, the endpoint death or transplantation occurred in 12 of 56 (21.4%) NTM‐positive patients and 92 of 198 (46.5%) NTM‐negative patients (odds ratio, 3.17; 95% CI, 1.53–7.00; *p* = 0.001). Among the 104 patients who reached the endpoint of death or transplantation, 103 proceeded to transplantation, with one death occurring before transplantation in the NTM‐negative group. Of all transplanted patients, eleven were diagnosed with NTM prior to transplant, and one was diagnosed post‐transplant (total of 12 [21.4%]), while 91 (46.0%) were from the NTM‐negative group (odds ratio, 3.11; 95% CI, 1.50–6.86; *p* = 0.001) (Table 1). The primary endpoint results were similar in the subgroup of MABSC‐positive patients only (Table S1).

### 3.3 Outcomes Post‐Transplantation

Out of the 103 patients who received a lung transplant, eleven patients were NTM‐positive before transplantation, eight patients had MABSC, and three patients had MAC. NTM eradication was achieved in eight (72.7%) patients at the time of transplantation. Among the 92 patients who were NTM‐negative pre‐transplantation, two (2.2%) had a subsequent positive NTM culture, with one patient meeting the criteria for NTM positivity. Characteristics of patients being either NTM‐negative or NTM‐positive before lung transplantation did not differ (Table 2).

**TABLE 2:** Demographics of patients having received lung transplantation and comparison of NTM‐positive to NTM‐negative cases (defined as at least two independent confirmed positive cultures of an identical NTM species).

	Overall *n* = 103	NTM negative *n* = 92	NTM positive (≥ 2 pos) *n* = 11	*p*‐value	HR	95% CI
Male (%)	51 (49.5)	46 (50.0)	5 (45.5)	1		
Median age (years, [IQR])	38.00 [31.50, 43.00]	38.00 [33.00, 43.00]	29.00 [26.00, 36.50]	0.076		
Mutation^a^ (%)				0.81		
Other mutation	7 (6.8)	6 (6.5)	1 (9.1)			
Heterozygous for F508del	24 (23.3)	20 (21.7)	4 (36.4)			
Homozygous for F508del	53 (51.5)	47 (51.1)	6 (54.5)			
Pancreatic insufficiency (%)	97 (94.2)	88 (95.7)	9 (81.8)	0.123		
Oral steroid treatment^b^ (%)	52 (50.5)	45 (48.9)	7 (63.6)	0.526		
Long‐term azithromycin treatment (%)	76 (73.8)	70 (76.1)	6 (54.4)	0.151		
Chronic *Pseudomonas aeruginosa* infection (%)	93 (90.3)	83 (90.2)	10 (90.9)	1		
*Burkholderia cepacia* complex infection (%)	14 (13.6)	11 (12.0)	3 (27.3)	0.17		
Death (%)	15 (14.6)	12 (13.0)	3 (27.3)	0.024	4.01	1.08–14.86
Median age at lung transplantation (years, [IQR])	25.00 [21.50, 32.00]	25.00 [21.75, 32.00]	25.00 [22.00, 31.50]	0.919		
Survival post lung transplantation (months, [IQR])	108.00 [66.50, 166.50]	110.50 [69.00, 170.50]	64.00 [39.50, 125.00]	0.032		
Re‐transplanted (%)	4 (3.9)	4 (4.3)	0 (0.0)	1		
CLAD (%)	43 (41.7)	40 (43.5)	3 (27.3)	0.35		
NTM‐associated complications post‐lung transplantation (%)						
Pulmonary infection, treated (%)	3 (2.9)	1 (1.1)	2 (18.2)	0.03		
Extrapulmonary infection, treated (%)	1 (1.0)	0 (0.0)	1 (9.1)	0.107		
Last FEV1 (%, [IQR])	75.00 [54.25, 89.00]	76.00 [55.00, 89.00]	71.00 [46.50, 86.00]	0.46		
NTM positive pre‐lung transplantation (%)	11 (10.7)	0 (0.0)	11 (100.0)			
NTM positive post lung transplantation (%)	5 (4.9)	2 (2.2)	3 (27.3)	0.008		
Chronic *Pseudomonas aeruginosa* infection post lung transplantation (%)	83 (80.6)	74 (80.4)	9 (81.8)	1		

Abbreviations: CF, cystic fibrosis; CLAD, chronic lung allograft dysfunction; FEV1, forced expiratory volume in one second;

NTM, nontuberculous mycobacteria.

^a^ Information on mutation was available in 84, 81.6% of patients.

^b^ Defined as at least one course of oral corticosteroid therapy administered at any time during the observation period.

**FIGURE 2:**
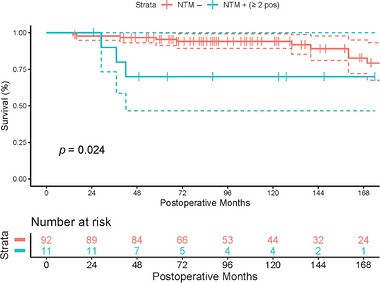
Survival after lung transplantation in NTM‐negative (NTM −) and NTM‐positive (NTM +) patients (defined as at least two independent confirmed positive cultures of an identical NTM species).

Mortality after lung transplantation was higher in the NTM‐positive cohort (counting only patients positive at the time of transplant), with death of any cause occurring in three patients (27.3%) compared to 12 (13.0%) NTM‐negative patients (hazard ratio, 4.01, 95% CI, 1.08–14.86; *p* = 0.024) (Figure 2). Median survival was 64 months (interquartile range, 39.5–125) for NTM‐positive and 110.50 months (interquartile range, 69–170.50) for NTM‐negative transplant recipients. Four patients of the NTM‐negative cohort required re‐transplantation, whereas none of the NTM‐positive patients did. After lung transplantation, two NTM‐positive patients and one NTM‐negative patient suffered from NTM‐associated pulmonary complications, and one patient developed an extrapulmonary infection with a fatal outcome. These results remained statistically significant when a more stringent definition of NTM‐positivity was applied (three independent positive cultures), or when only MABSC‐positive patients were compared to NTM‐negative patients (Figures S1 and S2).

Secondary outcome analysis did not reach statistical significance, with 40 patients (43.5%) from the NTM‐negative and three patients (27.3%) of the NTM‐positive cohort developing CLAD. Further, the last measured FEV1 appeared to be similar in both cohorts (Table 2). The analysis did not change when NTM‐negative patients were compared to MABSC‐positive patients only (Table S3).

## 4 Discussion

NTM infection has been associated with increased mortality as well as a greater risk of developing CLAD [7, 16] after lung transplantation. It has therefore been considered to be a relative or absolute contraindication for lung transplantation at many transplant centers. Whilst this study confirms previous findings regarding a higher mortality after lung transplantation in the NTM‐positive cohort, no association between NTM infection and CLAD development was observed. Furthermore, for CF‐positive patients, NTM‐positivity correlated with a lower risk of progression to death or transplantation.

The overall prevalence of NTM in CF patients treated at the Zurich and Lausanne CF centers was 20.7% when all patients who met the criteria of having at least two independent positive sputum samples of an identical NTM species were considered. A recent meta‐analysis including 95 non‐registry and registry studies reported a lower global prevalence of 7.9% [17], as did recent studies in Europe showing prevalence rates ranging from 1.3% in Italy to 11% in Scandinavia [3, 18, 19, 20, 21, 22]. A North American study conducted in 2018 reported a prevalence of 20% [23], which aligns more closely with our findings and reflects the rising incidence rates since 1990. Given that this study does not represent all age groups, slight variations in prevalence are to be expected. Concerning the increasing incidence rates, different surveillance strategies or newly established general screening protocols for NTM are often mentioned as a possible explanation [23, 24]. In addition, recent studies have focused on hospital‐based cross‐infection between CF patients [25, 26, 27, 28, 29], contrasting with the previously accepted notion that NTM acquisition occurred primarily through individual exposure to soil or water sources [30, 31]. However, the underlying cause of increasing incident rates of NTM remains poorly understood.

With a prevalence of 75.0%, MABSC was the most common species. This is in accordance with previous studies, indicating that MABSC is the predominant species in Europe [3, 19, 21, 22, 32], while MAC is more common in North America [23, 24]. Apart from environmental factors [33, 34], such as specific water‐quality constituents influencing the metabolism and growth of either MAC or MABSC [35], the reason for geographic variation in NTM species distribution remains elusive.

Previous studies indicated MABSC‐positive patients were younger, were more likely to be F508del homozygous, and had a higher prevalence of pancreatic insufficiency, characteristics often associated with a more severe CF phenotype [7, 36, 37]. Some of these trends were reinforced in this study, with MABSC‐positive patients still being younger and having acquired MABSC earlier in life than other species, even though this study did not include children under the age of 16. We did not compare MABSC to MAC directly, yet MAC was responsible for almost all remaining NTM cases. Regarding disease severity, our findings showed a trend toward a higher likelihood of pancreatic insufficiency among MABSC‐positive patients, consistent with the findings of a French case‐control study by Catherinot et al. [36]. In addition, co‐infection with other pathogens, such as chronic *P. aeruginosa* infection, appeared to be more common in MABSC and NTM‐positive patients in general, although none of these risk factors reached statistical significance. One possible explanation would be that more severe forms of CF are more susceptible not only to MABSC but to NTM infections in general. It is impossible to discern, however, whether chronic NTM infection, especially with MABSC, leads to lung function decline or is the consequence of a more advanced lung damage [3, 7, 10, 22]. In this study, NTM‐positive patients were less likely to experience death or transplantation, and this finding remained consistent when the subgroup of MABSC patients was analyzed separately. Given that ongoing NTM infection is not an absolute contraindication for lung transplantation at the Zurich and Lausanne CF center, the lower progression to death and transplantation, and specifically lung transplantation in the NTM‐positive cohort, cannot be attributed to a more conservative approach to transplantation. However, it is possible that ongoing NTM infection contributed to a combination of relative contraindications, ultimately reinforcing the clinical decision against lung transplantation. Another explanation contributing to the observed results could be that by not including all age groups in this study, the most severe cases affecting children under the age of 16 are possibly missing, and thus distort the prevalence of outcome death or transplantation. However, due to the small sample size, our results have to be interpreted with caution. Also, all data analyzed in this study were collected in the pre‐CFTR modulator era, which may limit comparability to the current CF patient population.

In this study, macrolide maintenance therapy was significantly associated with NTM‐negativity, suggesting a reduced risk of NTM acquisition. While these findings are in line with two case‐control studies [38, 39] and one retrospective cohort study [40], there are also previous single‐center studies considering chronic macrolide therapy as a potential risk factor for NTM infection [41, 42], leaving the role of macrolide maintenance therapy in NTM acquisition uncertain. Notably, macrolide immunomodulatory monotherapy has been linked to inducible macrolide resistance in NTM, mediated by the novel erythromycin ribosomal methylase gene, *erm*(41) [43].

This specific resistance pattern may have therapeutic implications for future antibiotic NTM treatments. As patients with macrolide‐resistant NTM tend to have poorer outcomes, the 2016 CF Foundation and European Cystic Fibrosis Society guidelines recommend discontinuing macrolide monotherapy following a single NTM‐positive culture [44].

The association between NTM infection and mortality after lung transplantation has been inconsistently reported in the literature. A single‐center study by Esther et al. [7] initially suggested that NTM infection post lung transplantation was associated with increased mortality and a higher risk of developing CLAD. Subsequent studies supported the finding of increased all‐cause mortality but did not observe a significant association with the development of BOS [16, 45]. Notably, these studies were not exclusively conducted for CF patients. However, various case reports and single‐center studies have recently reported acceptable short and long‐term outcomes after lung transplantation in patients with MABSC infection [46, 47, 48, 49, 50, 51, 52]. Our study showed a statistically significant decrease in the median survival in NTM‐positive patients after lung transplantation. Nevertheless, no association was observed between NTM infection and the development of CLAD, and the last measured FEV1 was comparable between the two cohorts.

Complications related to NTM infection were observed in two NTM‐positive patients, both of whom developed pulmonary infection with MABSC, and one patient suffered additionally from a chest wall abscess. Only one NTM‐negative patient developed a pulmonary infection with MAC with a fatal outcome after lung transplantation. Previous case series reported a complication rate of 23%–75% [46, 50, 52], indicating increased morbidity associated with NTM infection post‐transplantation. Nearly all patients infected with NTM prior to or after lung transplantation received NTM‐specific treatment. However, there are no established guidelines on treatment duration before or after lung transplantation. Considering that post‐transplant infection was, apart from one post‐transplant infection with MAC, restricted to those patients with pre‐transplant MABSC isolates, and patients who achieved long‐term sputum conversion prior to transplantation did not show reinfection after transplantation, pre‐transplant eradication is probably a worthwhile goal to be attempted. However, distinguishing between NTM infection and mere colonization seems to be crucial, as several patients in our study with subsequent MABSC isolates before or after transplantation did not show a difference in survival or development of CLAD despite not receiving antibiotic treatment. Observation with close surveillance may be a viable approach for these patients.

This analysis has several limitations. Despite a sizable cohort of CF lung transplant recipients, the sample size of NTM‐positive patients remained very small. In addition, NTM infection was primarily determined based on microbiological isolation, without accounting for clinical or radiological manifestations. Because such manifestations are common but non‐specific for CF patients, distinguishing between colonization and disease remains difficult. This may have led to an overestimation of the true incidence of NTM infection and potentially influenced the outcomes. Furthermore, our study exclusively included patients aged 16 years or older, limiting the applicability of the findings to individuals under 16, a group at high risk for MABSC infection.

Despite its limitations, this study contributes to the ongoing discussion regarding the impact of NTM infections on CF patients, both in general and specifically in the context of lung transplantation outcomes. Notably, as one of the few multi‐center studies and among the largest of its kind, it provides valuable evidence demonstrating that favorable outcomes after lung transplantation in NTM‐positive patients are achievable. Thus, lung transplantation should not be considered an absolute contraindication. However, given the risk of serious complications, careful single‐patient assessment is essential, and individualized management and surveillance protocols should be implemented both before and after transplantation.

## Author Contributions

**Shirin Barcikowski**: conceptualization, data curation, formal analysis, writing – original draft, writing – review and editing. **Ribal Bou Mjahed**: data curation. **Oriol Manuel**: writing – review and editing. **Macé M. Schuurmans**: writing – review and editing. **Adrian Alonso**: data curation. **Zisis Balmpouzis**: writing – review and editing. **Jesica Mazza‐Stalder**: writing – review and editing. **Angela Koutsokera**: writing – review and editing. **Nicolas J. Mueller**: conceptualization, writing – review and editing.

## Conflicts of Interest

The authors declare no conflicts of interest.

## Supporting information




**Supporting File 1**: tid70190‐sup‐0001‐Figures.docx

**Supporting File 2**: tid70190‐sup‐0002‐Tables.docx

**Supporting File 3**: Visual Abstract

## References

[1] D. E. Griffith , T. Aksamit , B. A. Brown‐Elliott , et al., “An Official ATS/IDSA Statement: Diagnosis, Treatment, and Prevention of Nontuberculous Mycobacterial Diseases,” American Journal of Respiratory and Critical Care Medicine 175, no. 4 (2007): 367–416, 10.1164/rccm.200604-571ST. 17277290

[2] J. M. Leung and K. N. Olivier , “Nontuberculous Mycobacteria,” Current Opinion in Pulmonary Medicine 19, no. 6 (2013): 662–669, 10.1097/MCP.0b013e328365ab33. 24048085 PMC6684957

[3] T. Qvist , M. Gilljam , B. Jönsson , et al., “Epidemiology of Nontuberculous Mycobacteria Among Patients With Cystic Fibrosis in Scandinavia,” Journal of Cystic Fibrosis 14, no. 1 (2015): 46–52, 10.1016/j.jcf.2014.08.002. 25178871 PMC4298356

[4] T. Qvist , T. Pressler , N. Høiby , and T. L. Katzenstein , “Shifting Paradigms of Nontuberculous Mycobacteria in Cystic Fibrosis,” Respiratory Research 15, no. 1 (2014): 41, 10.1186/1465-9921-15-41. 24725650 PMC3986433

[5] S. C. Leao , E. Tortoli , J. P. Euzéby , and M. J. Garcia , “Proposal That *Mycobacterium massiliense* and *Mycobacterium bolletii* be United and Reclassified as *Mycobacterium abscessus* subsp. *bolletii* comb. nov., Designation of *Mycobacterium abscessus* subsp. *abscessus* subsp. nov. And Emended Description of *Mycobacterium abscessus* ,” International Journal of Systematic and Evolutionary Microbiology 61 (2011): 2311–2313. 21037035 10.1099/ijs.0.023770-0

[6] T. Adekambi , “rpoB Gene Sequence‐Based Characterization of Emerging Non‐Tuberculous Mycobacteria With Descriptions of *Mycobacterium bolletii* sp. nov., *Mycobacterium phocaicum* sp. nov. And *Mycobacterium aubagnense* sp. nov,” International Journal of Systematic and Evolutionary Microbiology 56 (2006): 133–143, 10.1099/ijs.0.63969-0. 16403878

[7] C. R. Esther , D. A. Esserman , P. Gilligan , A. Kerr , and P. G. Noone , “Chronic *Mycobacterium abscessus* Infection and Lung Function Decline in Cystic Fibrosis,” Journal of Cystic Fibrosis 9, no. 2 (2010): 117–123, 10.1016/j.jcf.2009.12.001. 20071249 PMC3837580

[8] J. Van Ingen , B. E. Ferro , W. Hoefsloot , M. J. Boeree , and D. Van Soolingen , “Drug Treatment of Pulmonary Nontuberculous Mycobacterial Disease in HIV‐Negative Patients: The Evidence,” Expert Review of Anti‐Infective Therapy 11, no. 10 (2013): 1065–1077, 10.1586/14787210.2013.830413. 24124798

[9] G. J. Ballarino , K. N. Olivier , R. J. Claypool , S. M. Holland , and D. R. Prevots , “Pulmonary Nontuberculous Mycobacterial Infections: Antibiotic Treatment and Associated Costs,” Respiratory Medicine 103, no. 10 (2009): 1448–1455, 10.1016/j.rmed.2009.04.026. 19467851 PMC2739259

[10] S. L. Martiniano , M. K. Sontag , C. L. Daley , J. A. Nick , and S. D. Sagel , “Clinical Significance of a First Positive Nontuberculous Mycobacteria Culture in Cystic Fibrosis,” Annals of the American Thoracic Society 11, no. 1 (2014): 36–44, 10.1513/AnnalsATS.201309-310OC. 24251858 PMC3972987

[11] “2024‐Patient‐Registry‐Highlights‐Report,” Cystic Fibrosis Foundation , accessed June 19, 2025, https://www.cff.org/sites/default/files/2025‐05/2024‐Patient‐Registry‐Highlights‐Report.pdf.

[12] A. Tissot , M. F. Thomas , P. A. Corris , and M. Brodlie , “NonTuberculous Mycobacteria Infection and Lung Transplantation in Cystic Fibrosis: A Worldwide Survey of Clinical Practice,” BMC Pulmonary Medicine 18, no. 1 (2018): 86, 10.1186/s12890-018-0635-3. 29788939 PMC5964879

[13] M.‐L. Luong , Y. Nakamachi , F. P. Silveira , et al., “Management of Infectious Disease Syndromes in Thoracic Organ Transplants and Mechanical Circulatory Device Recipients: A Delphi Panel,” Transplant Infectious Disease 26, no. 3 (2024): e14251, 10.1111/tid.14251. 38351512

[14] M. M. Brett , E. J. Simmonds , A. T. Ghoneim , and J. M. Littlewood , “The Value of Serum IgG Titres Against *Pseudomonas aeruginosa* in the Management of Early Pseudomonal Infection in Cystic Fibrosis,” Archives of Disease in Childhood 67, no. 9 (1992): 1086–1088, 10.1136/adc.67.9.1086. 1417051 PMC1793633

[15] G. L. Woods , B. A. Brown‐Elliott , P. S. Conville , et al., Susceptibility Testing of Mycobacteria, Nocardiae, and Other Aerobic Actinomycetes, 2nd ed. (Clinical and Laboratory Standards Institute, 2011). 31339680

[16] H. C. Huang , S. S. Weigt , A. Derhovanessian , et al., “Non‐Tuberculous Mycobacterium Infection After Lung Transplantation Is Associated With Increased Mortality,” Journal of Heart and Lung Transplantation 30, no. 7 (2011): 790–798, 10.1016/j.healun.2011.02.007. PMC3942162 21482148

[17] M. D. Prieto , M. E. Alam , A. N. Franciosi , and B. S. Quon , “Global Burden of Nontuberculous Mycobacteria in the Cystic Fibrosis Population: A Systematic Review and Meta‐Analysis,” ERJ Open Research 9, no. 1 (2023): 00336–2022, https://www.ncbi.nlm.nih.gov/pubmed/36605902. 10.1183/23120541.00336-2022 PMC9808535 36605902

[18] B. Giordani , A. Amato , F. Majo , et al., “Italian Cystic Fibrosis Registry (ICFR). Report 2019–2020,”Epidemiologia E Prevenzione 43, no. 4S1 (2022): 1–36. 10.19191/EP19.4.S1.067 31370382

[19] M. Steindor , S. Hafkemeyer , C. Ruckes , F. Stehling , L. Naehrlich , and F. C. Ringshausen , “Epidemiological Trends in Nontuberculous Mycobacterial Infection Among People With Cystic Fibrosis in Germany,” International Journal of Infectious Diseases 129 (2023): 32–39, 10.1016/j.ijid.2023.01.032. 36736578

[20] A. I. Gardner , E. Mcclenaghan , G. Saint , P. S. Mcnamara , M. Brodlie , and M. F. Thomas , “Epidemiology of Nontuberculous Mycobacteria Infection in Children and Young People With Cystic Fibrosis: Analysis of UK Cystic Fibrosis Registry,” Clinical Infectious Diseases 68, no. 5 (2019): 731–737, 10.1093/cid/ciy531. 29982302 PMC6376093

[21] A.‐L. Roux , E. Catherinot , F. Ripoll , et al., “Multicenter Study of Prevalence of Nontuberculous Mycobacteria in Patients With Cystic Fibrosis in France,” Journal of Clinical Microbiology 47, no. 12 (2009): 4124–4128, 10.1128/JCM.01257-09. 19846643 PMC2786646

[22] D. Zomer , J. Van Ingen , R. Hofland , et al., “Epidemiology and Management of Nontuberculous Mycobacterial Disease in People With Cystic Fibrosis, the Netherlands,” Journal of Cystic Fibrosis 22, no. 2 (2023): 327–333, 10.1016/j.jcf.2022.10.009. 36347785

[23] J. Adjemian , K. N. Olivier , and D. R. Prevots , “Epidemiology of Pulmonary Nontuberculous Mycobacterial Sputum Positivity in Patients With Cystic Fibrosis in the United States, 2010–2014,” Annals of the American Thoracic Society 15, no. 7 (2018): 817–826. 29897781 10.1513/AnnalsATS.201709-727OC PMC6137684

[24] K. N. Olivier , D. J. Weber , R. J. Wallace , et al., “Nontuberculous Mycobacteria,” American Journal of Respiratory and Critical Care Medicine 167, no. 6 (2003): 828–834, 10.1164/rccm.200207-678OC. 12433668

[25] C. Ruis , J. M. Bryant , S. C. Bell , et al., “Dissemination of *Mycobacterium abscessus* via Global Transmission Networks,” Nature Microbiology 6, no. 10 (2021): 1279–1288, 10.1038/s41564-021-00963-3. PMC8478660 34545208

[26] J. M. Bryant , D. M. Grogono , D. Rodriguez‐Rincon , et al., “Emergence and Spread of a Human‐Transmissible Multidrug‐Resistant Nontuberculous Mycobacterium,” Science 354, no. 6313 (2016): 751–757, 10.1126/science.aaf8156. 27846606 PMC5142603

[27] J. M. Bryant , D. M. Grogono , D. Greaves , et al., “Whole‐Genome Sequencing to Identify Transmission of *Mycobacterium abscessus* Between Patients With Cystic Fibrosis: A Retrospective Cohort Study,” Lancet 381, no. 9877 (2013): 1551–1560, 10.1016/S0140-6736(13)60632-7. 23541540 PMC3664974

[28] M. L. Aitken , A. Limaye , P. Pottinger , et al., “Respiratory Outbreak of *Mycobacterium abscessus* Subspecies Massiliense in a Lung Transplant and Cystic Fibrosis Center,” American Journal of Respiratory and Critical Care Medicine 185, no. 2 (2012): 231–232, 10.1164/ajrccm.185.2.231. 22246710

[29] J. Yan , A. Kevat , E. Martinez , et al., “Investigating Transmission of *Mycobacterium abscessus* amongst Children in an Australian Cystic Fibrosis Centre,” Journal of Cystic Fibrosis 19, no. 2 (2020): 219–224, 10.1016/j.jcf.2019.02.011. 30853372

[30] T. P. Primm , C. A. Lucero , and J. O. Falkinham , “Health Impacts of Environmental Mycobacteria,” Clinical Microbiology Reviews 17, no. 1 (2004): 98–106, 10.1128/CMR.17.1.98-106.2004. 14726457 PMC321467

[31] J. O. Falkinham , “Nontuberculous Mycobacteria From Household Plumbing of Patients With Nontuberculous Mycobacteria Disease,” Emerging Infectious Diseases 17, no. 3 (2011): 419–424. 21392432 10.3201/eid1703.101510 PMC3166028

[32] S. Lipworth , N. Hough , N. Weston , et al., “Epidemiology of *Mycobacterium abscessus* in England: An Observational Study,” Lancet Microbe 2, no. 10 (2021): e498–e507, 10.1016/S2666-5247(21)00128-2. 34632432 PMC8481905

[33] M. J. Gebert , M. Delgado‐Baquerizo , A. M. Oliverio , et al., “Ecological Analyses of Mycobacteria in Showerhead Biofilms and Their Relevance to Human Health,” mBio 9, no. 5 (2018), 10.1128/mBio.01614-18. PMC6212831 30377276

[34] C. L. Tzou , M. A. Dirac , A. L. Becker , et al., “Association Between *Mycobacterium avium* Complex Pulmonary Disease and Mycobacteria in Home Water and Soil,” Annals of the American Thoracic Society 17, no. 1 (2020): 57–62. 31644315 10.1513/AnnalsATS.201812-915OC PMC6944351

[35] E. M. Lipner , J. P. French , R. A. Mercaldo , et al., “The Risk of Pulmonary NTM Infections and Water‐Quality Constituents Among Persons With Cystic Fibrosis in the United States, 2010–2019,” Environmental Epidemiology 7, no. 5: e266. 10.1097/EE9.0000000000000266 PMC10569765 37840858

[36] E. Catherinot , A.‐L. Roux , M.‐A. Vibet , et al., “ *Mycobacterium avium* and *Mycobacterium abscessus* Complex Target Distinct Cystic Fibrosis Patient Subpopulations,” Journal of Cystic Fibrosis 12, no. 1 (2013): 74–80, 10.1016/j.jcf.2012.06.009. 22857820

[37] C. Pierre‐Audigier , A. S. Ferroni , I. Sermet‐Gaudelus , et al., “Age‐Related Prevalence and Distribution of Nontuberculous Mycobacterial Species Among Patients With Cystic Fibrosis,” Journal of Clinical Microbiology 43, no. 7 (2005): 3467–3470, 10.1128/JCM.43.7.3467-3470.2005. 16000480 PMC1169165

[38] N. Coolen , P. Morand , C. Martin , et al., “Reduced Risk of Nontuberculous Mycobacteria in Cystic Fibrosis Adults Receiving Long‐Term Azithromycin,” Journal of Cystic Fibrosis 14, no. 5 (2015): 594–599, 10.1016/j.jcf.2015.02.006. 25735458

[39] A. M. Binder , J. Adjemian , K. N. Olivier , and D. R. Prevots , “Epidemiology of Nontuberculous Mycobacterial Infections and Associated Chronic Macrolide Use Among Persons With Cystic Fibrosis,” American Journal of Respiratory and Critical Care Medicine 188, no. 7 (2013): 807–812, 10.1164/rccm.201307-1200OC. 23927602 PMC3826274

[40] J. D. Cogen , F. Onchiri , J. Emerson , et al., “Chronic Azithromycin Use in Cystic Fibrosis and Risk of Treatment‐Emergent Respiratory Pathogens,” Annals of the American Thoracic Society 15, no. 6 (2018): 702–709, 10.1513/AnnalsATS.201801-012OC. 29474110 PMC6850787

[41] I. Levy , G. Grisaru‐Soen , L. Lerner‐Geva , et al., “Multicenter Cross‐Sectional Study of Nontuberculous Mycobacterial Infections Among Cystic Fibrosis Patients, Israel,” Emerging Infectious Diseases 14, no. 3 (2008): 378–384, 10.3201/eid1403.061405. 18325250 PMC2570835

[42] M. Renna , C. Schaffner , K. Brown , et al., “Azithromycin Blocks Autophagy and May Predispose Cystic Fibrosis Patients to Mycobacterial Infection,” Journal of Clinical Investigation 121, no. 9 (2011): 3554–3563, 10.1172/JCI46095. 21804191 PMC3163956

[43] K. A. Nash , B. A. Brown‐Elliott , and R. J. Wallace , “A Novel Gene, *erm*(41), Confers Inducible Macrolide Resistance to Clinical Isolates of *Mycobacterium abscessus* but Is Absent From *Mycobacterium chelonae* ,” Antimicrobial Agents and Chemotherapy 53, no. 4 (2009): 1367–1376, 10.1128/AAC.01275-08. 19171799 PMC2663066

[44] R. A. Floto , K. N. Olivier , L. Saiman , et al., “US Cystic Fibrosis Foundation and European Cystic Fibrosis Society Consensus Recommendations for the Management of Non‐Tuberculous Mycobacteria in Individuals With Cystic Fibrosis,” Thorax 71, no. S1 (2016): i1–i22, 10.1136/thoraxjnl-2015-207360. 26666259 PMC4717371

[45] S. A. Longworth , E. A. Blumberg , T. D. Barton , and C. Vinnard , “Non‐Tuberculous Mycobacterial Infections After Solid Organ Transplantation: A Survival Analysis,” Clinical Microbiology and Infection 21, no. 1 (2015): 43–47. 25636926 10.1016/j.cmi.2014.07.001 PMC4313615

[46] M. Osmani , D. Sotello , S. Alvarez , J. A. Odell , and M. Thomas , “ *Mycobacterium abscessus* Infections in Lung Transplant Recipients: 15‐Year Experience From a Single Institution,” Transplant Infectious Disease 20, no. 2 (2018): e12835, 10.1111/tid.12835. 29359872

[47] S. K. Shah , K. J. Mcanally , L. Seoane , et al., “Analysis of Pulmonary Non‐Tuberculous Mycobacterial Infections After Lung Transplantation,” Transplant Infectious Disease 18, no. 4 (2016): 585–591, 10.1111/tid.12546. 27368989

[48] T. Hirama , L. G. Singer , S. K. Brode , T. K. Marras , and S. Husain , “Outcomes of a Peri‐ and Postoperative Management Protocol for Non‐TB Mycobacteria in Lung Transplant Recipients,” Chest 158, no. 2 (2020): 523–528, 10.1016/j.chest.2020.01.056. 32247715

[49] A. A. Perez , J. P. Singer , B. S. Schwartz , et al., “Management and Clinical Outcomes After Lung Transplantation in Patients With Pre‐Transplant *Mycobacterium abscessus* Infection: A Single Center Experience,” Transplant Infectious Disease 21, no. 3 (2019): e13084, 10.1111/tid.13084. 30924986

[50] T. Qvist , T. Pressler , V. O. Thomsen , M. Skov , M. Iversen , and T. L. Katzenstein , “Nontuberculous Mycobacterial Disease Is Not a Contraindication to Lung Transplantation in Patients With Cystic Fibrosis: A Retrospective Analysis in a Danish Patient Population,” Transplantation Proceedings 45, no. 1 (2013): 342–345, 10.1016/j.transproceed.2012.02.035. 23267788

[51] D. Raats , N. Lorent , V. Saegeman , et al., “Successful Lung Transplantation for Chronic *Mycobacterium abscessus* Infection in Advanced Cystic Fibrosis, a Case Series,” Transplant Infectious Disease 21, no. 2 (2019): e13046, 10.1111/tid.13046. 30597699

[52] L. J. Lobo , L. C. Chang , C. R. Esther , P. H. Gilligan , Z. Tulu , and P. G. Noone , “Lung Transplant Outcomes in Cystic Fibrosis Patients With Pre‐Operative *Mycobacterium abscessus* Respiratory Infections,” Clinical Transplantation 27, no. 4 (2013): 523–529, 10.1111/ctr.12140. 23710571

