# Supplementary material for: The Role of Nontuberculous Mycobacteria in Patients With Cystic Fibrosis Advanced Lung Disease
Source: Transpl Infect Dis. 2026 Feb 25;28(3):e70190. doi: 10.1111/tid.70190 (PMC13262555; doi:10.1111/tid.70190)
Supplement: Supplementary file 1 — Supporting File 1: tid70190‐sup‐0001‐Figures.docx [file TID-28-e70190-s002.docx]

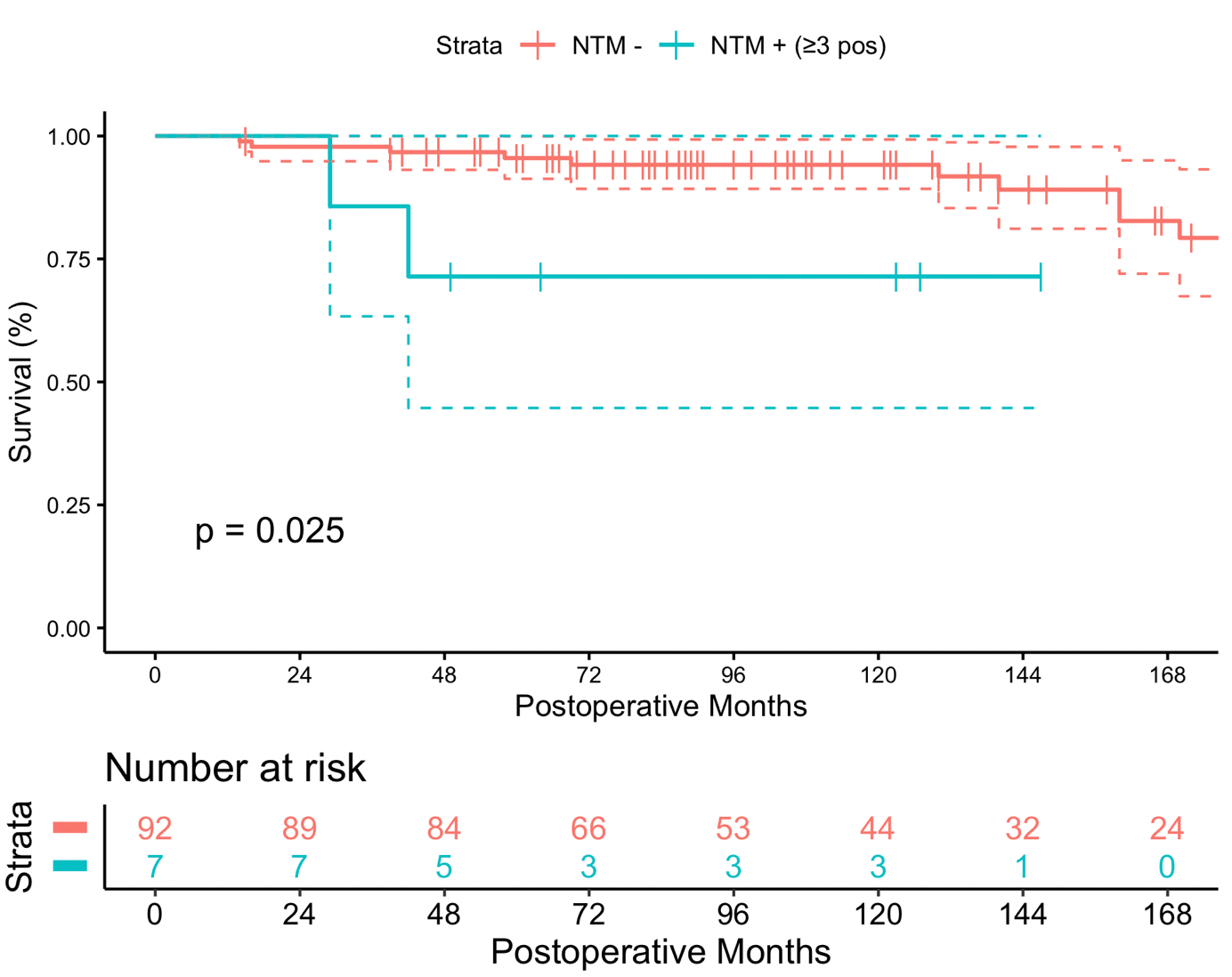


Supplementary Figure 1: **Survival after lung transplantation** in NTM-negative (NTM -) and NTM-positive (NTM +) patients (defined as at least three independent confirmed positive cultures of an identical NTM species).


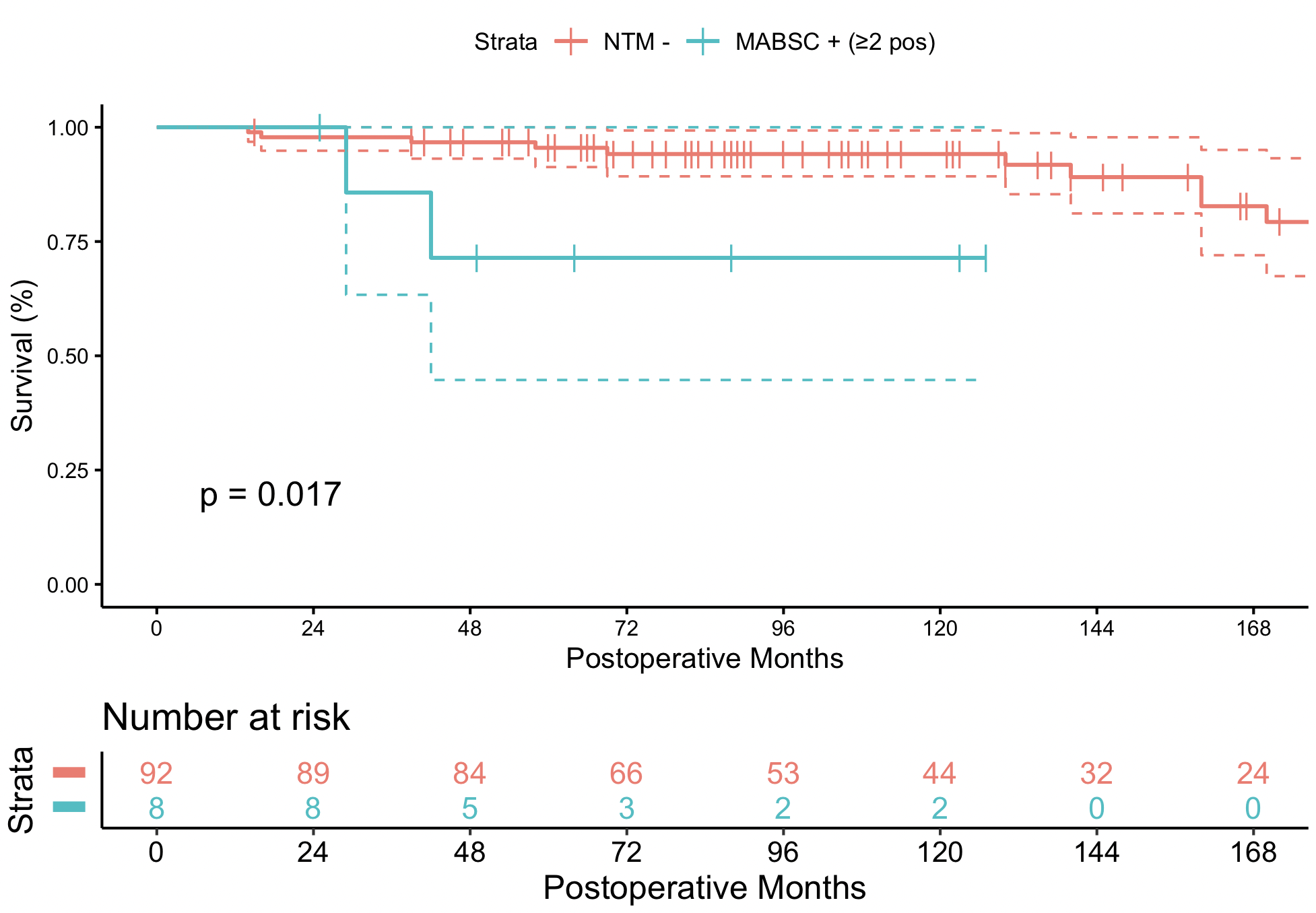


Supplementary Figure 2: **Survival after lung transplantation** in NTM-negative (NTM -) and MABSC-positive (MABSC +) patients (defined as at least two independent confirmed positive cultures).
