# Supplementary material for: The Role of Nontuberculous Mycobacteria in Patients With Cystic Fibrosis Advanced Lung Disease
Source: Transpl Infect Dis. 2026 Feb 25;28(3):e70190. doi: 10.1111/tid.70190 (PMC13262555; doi:10.1111/tid.70190)
Supplement: Supplementary file 2 — Supporting File 2: tid70190‐sup‐0002‐Tables.docx [file TID-28-e70190-s001.docx]

|  | NTM negative  n = 198 | MABSC positive (≥ 2)  n = 39 | p-Value | OR | 95% CI |
| --- | --- | --- | --- | --- | --- |
| Male (%) | 103 (52.0) | 23 (59.0) | 0.535 |  |  |
| Median age (years, [IQR]) | 33.00 [26.00, 41.00] | 28.00 [24.50, 36.50] | 0.057 |  |  |
| Mutation^†^ (%) |  |  | 0.233 |  |  |
| other mutation | 26 (13.1) | 2 (5.1) |  |  |  |
| heterozygous for F508del | 70 (35.4) | 19 (48.7) |  |  |  |
| homozygous for F508del | 82 (41.4) | 18 (46.2) |  |  |  |
| Transplanted (%) | 91 (46.0) | 8 (20.5) | 0.006 | 3.33 | 1.45–7.69 |
| Pancreatic insufficiency (%) | 173 (87.4) | 37 (94.9) | 0.284 |  |  |
| Oral steroid treatment^‡^ (%) | 69 (34.8) | 10 (25.6) | 0.353 |  |  |
| Chronic Pseudomonas aeruginosa infection (%) | 149 (75.3) | 32 (82.1) | 0.479 |  |  |
| Burkholderia cepacia complex infection (%) | 19 (9.6) | 6 (15.4) | 0.265 |  |  |
| Advanced lung disease (%) | 92 (46.5) | 8 (20.5) | 0.009 | 3.37 | 1.46 – 7.75 |
| Death pre lung transplantation (%) | 1 (0.5) | 0 (0.0) | 1 |  |  |
| Death pre and post lung transplantation (%) | 13 (6.6) | 2 (5.1) | 0.7 | 1.3 | 0.28-6.00 |

**Supplementary Table 1:** Demographics of MABSC-positive patients compared to NTM-negative patients (defined as at least two independent confirmed positive cultures of MABSC); ^†^ information on mutation was available in 217, 91.6% of patients; ^‡^ defined as at least one course of oral corticosteroid therapy administered at any time during the observation period. Abbreviations: MABSC, *M. abscessus* *complex*; NTM, nontuberculous mycobacteria; CF, cystic fibrosis.

|  | Non-MABSC positive (≥ 2)  n = 17 | MABSC positive (≥ 2)  n = 28 | p-Value | OR | 95% CI |
| --- | --- | --- | --- | --- | --- |
| Male (%) | 10 (58.8) | 16 (57.1) | 1 |  |  |
| Median age (years, [IQR]) | 41.00 [32.00, 54.00] | 27.00 [24.00, 38.25] | 0.008 |  |  |
| Mutation (%) |  |  | 0.565 |  |  |
| other mutation | 3 (17.6) | 2 (7.1) |  |  |  |
| heterozygous for F508del | 6 (35.3) | 13 (46.4) |  |  |  |
| homozygous for F508del | 8 (47.1) | 13 (46.4) |  |  |  |
| Transplanted (%) | 4 (23.5) | 8 (28.6) | 0.78 | 1.30 | 0.32-5.20 |
| Pancreatic insufficiency (%) | 13 (76.5) | 27 (96.4) | 0.115 |  |  |
| Oral steroid treatment^‡^ (%) | 4 (23.5) | 9 (32.1) | 0.78 |  |  |
| Chronic Pseudomonas aeruginosa infection (%) | 15 (88.2) | 26 (92.9) | 1 |  |  |
| Burkholderia cepacia complex infection (%) | 0 (0.0) | 4 (14.3) | 0.281 |  |  |
| Advanced lung disease (%) | 4 (23.5) | 8 (28.6) | 0.78 | 1.30 | 0.32-5.20 |
| Median age at first NTM positive culture (years, [IQR]) | 34.00 [25.00, 39.00] | 19.5 [16.75, 32.75] | 0.015 |  |  |
| Death pre and post lung transplantation (%) | 1 (5.9) | 2 (7.1) | 1 | 1.23 | 0.10-14.65 |

**Supplementary Table 2:** Demographics of Non-MABSC positive patients compared to MABSC positive patients (defined as at least two independent confirmed positive cultures of an NTM species); ^‡^ defined as at least one course of oral corticosteroid therapy administered at any time during the observation period. Abbreviations: MABSC, *M. abscessus* *complex*; NTM, nontuberculous mycobacteria; CF, cystic fibrosis.

|  | NTM negative  n = 92 | MABSC positive  (≥ 2 pos)  n = 8 | p-Value | HR | 95% CI |
| --- | --- | --- | --- | --- | --- |
| Male (%) | 46 (50.0) | 5 (50.0) | 1 |  |  |
| Median age (years, [IQR]) | 38.00 [33.00, 43.00] | 28.00 [26.00, 35.00] | 0.008 |  |  |
| Mutation^†^ (%) |  |  | 0.34 |  |  |
| other mutation | 6 (6.5) | 0 (0.0) |  |  |  |
| heterozygous for F508del | 20 (21.7) | 4 (50.0) |  |  |  |
| homozygous for F508del | 47 (51.1) | 4 (50.0) |  |  |  |
| Pancreatic insufficiency (%) | 88 (95.7) | 7 (87.5) | 0.347 |  |  |
| Oral steroid treatment^‡^ (%) | 45 (48.9) | 6 (75.0) | 0.269 |  |  |
| Long-term azithromycin treatment (%) | 70 (76.1) | 4 (50.0) | 0.2 |  |  |
| Chronic Pseudomonas aeruginosa infection (%) | 83 (90.2) | 7 (87.5) | 0.583 |  |  |
| Burkholderia cepacia complex infection (%) | 11 (12.0) | 3 (37.5) | 0.081 |  |  |
| At least one NTM positive culture (%) | 2 (2.2) | 11 (100.0) |  |  |  |
| Median age at first NTM positive culture (years, [IQR]) | 31.50 [29.75, 33.25] | 19.00 [16.00, 22.50] | 0.049 |  |  |
| Death (%) | 12 (13.0) | 2 (25.0) | 0.02 | 5.88 | 1.13- 30.59 |
| Median age at lung transplantation (years, [IQR]) | 25.00 [21.75, 32.00] | 24.50 [22.50, 27.25] | 0.563 |  |  |
| Survival post lung transplantation (months, [IQR]) | 110.50 [69.00, 170.50] | 56.50 [38.75, 96.75] | 0.013 |  |  |
| Re-transplanted (%) | 4 (4.3) | 0 (0.0) | 1 |  |  |
| CLAD (%) | 40 (43.5) | 2 (25.0) | 0.47 |  |  |
| NTM associated complications post lung transplantation (%) |  |  |  |  |  |
| Pulmonary infection, treated (%) | 1 (1.1) | 2 (25.0) | 0.016 |  |  |
| Extrapulmonary infection, treated (%) | 0 (0.0) | 1 (12.5) | 0.08 |  |  |
| Last FEV1 (%, [IQR]) | 76.00 [55.00, 89.00] | 71.00 [52.00, 80.50] | 0.621 |  |  |
| NTM positive pre lung transplantation (%) | 0 (0.0) | 8 (100.0) |  |  |  |
| NTM positive post lung transplantation (%) | 2 (2.2) | 3 (37.5) | 0.003 |  |  |
| Chronic Pseudomonas aeruginosa infection post lung transplantation (%) | 74 (80.4) | 6 (75.0) | 0.659 |  |  |

**Supplementary Table 3:** Demographics of patients having received lung transplantation and comparison of MABSC-positive to NTM-negative cases (defined as at least two independent confirmed positive cultures of MABSC); ^†^ information on mutation was available in 81, 81.0% of patients; ^‡^ defined as at least one course of oral corticosteroid therapy administered at any time during the observation period. Abbreviations: MABSC, *M. abscessus* *complex*, NTM, nontuberculous mycobacteria; CF, cystic fibrosis; CLAD, chronic lung allograft dysfunction; FEV1, forced expiratory volume in one second.
