# Supplementary material for: The Role of Nontuberculous Mycobacteria in Patients With Cystic Fibrosis Advanced Lung Disease
Source: Transpl Infect Dis. 2026 Feb 25;28(3):e70190. doi: 10.1111/tid.70190 (PMC13262555; doi:10.1111/tid.70190)
Supplement: Supplementary file 3 — Supporting File 3: Visual Abstract [file TID-28-e70190-s003.pptx]

## Slide 1
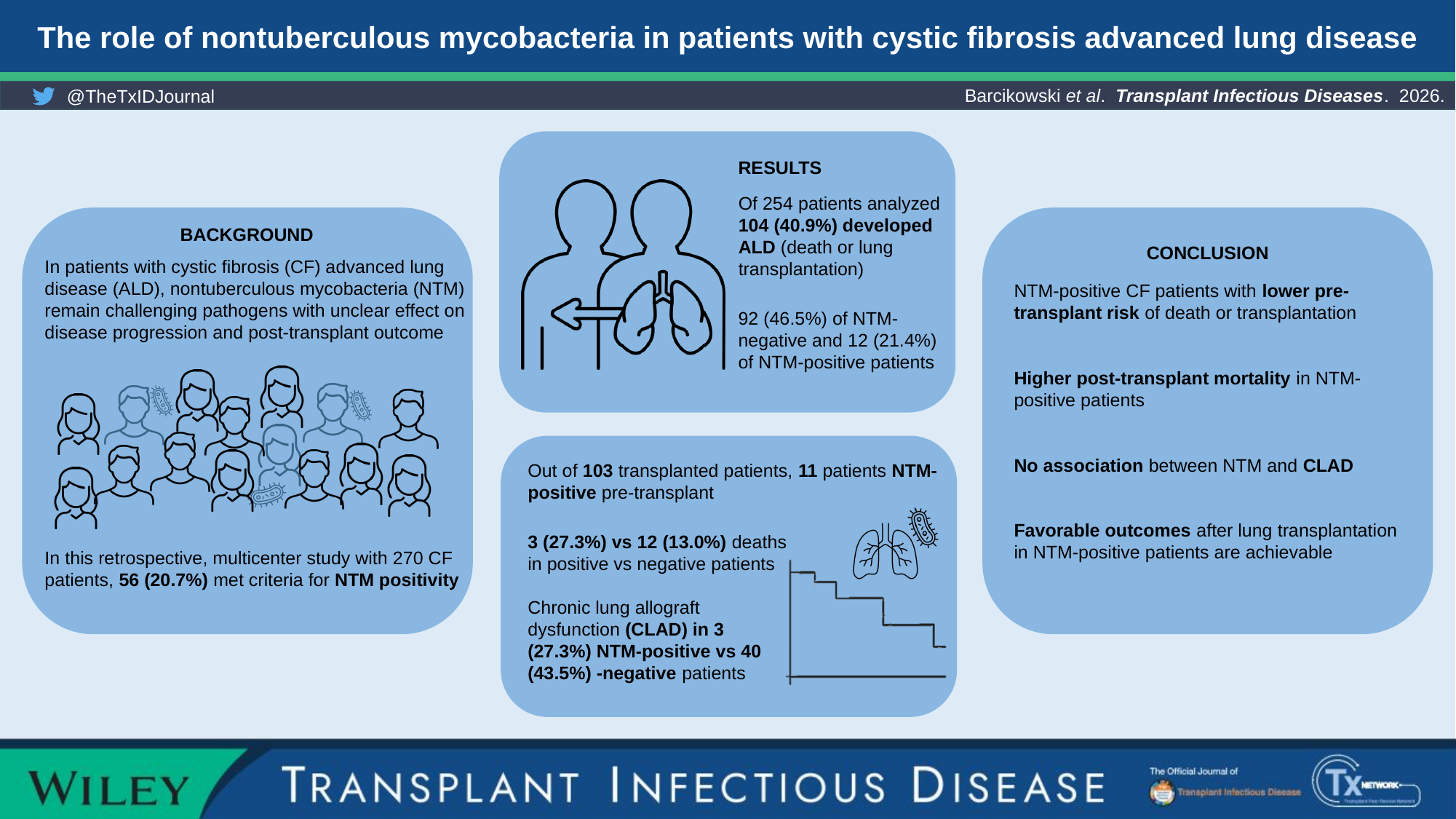

The role of nontuberculous mycobacteria in patients with cystic fibrosis advanced lung disease
Barcikowski et al. Transplant Infectious Diseases. 2026.
 @TheTxIDJournal
RESULTS
Of 254 patients analyzed 104 (40.9%) developed ALD (death or lung transplantation)
NTM-positive CF patients with lower pre-transplant risk of death or transplantation
Higher post-transplant mortality in NTM-positive patients
No association between NTM and CLAD
Favorable outcomes after lung transplantation in NTM-positive patients are achievable
BACKGROUND
CONCLUSION
In patients with cystic fibrosis (CF) advanced lung disease (ALD), nontuberculous mycobacteria (NTM) remain challenging pathogens with unclear effect on disease progression and post-transplant outcome
92 (46.5%) of NTM-negative and 12 (21.4%) of NTM-positive patients
Out of 103 transplanted patients, 11 patients NTM-positive pre-transplant
In this retrospective, multicenter study with 270 CF patients, 56 (20.7%) met criteria for NTM positivity
3 (27.3%) vs 12 (13.0%) deaths in positive vs negative patients
Chronic lung allograft dysfunction (CLAD) in 3 (27.3%) NTM-positive vs 40 (43.5%) -negative patients
